# Cost analysis of an innovative eHealth program in Nigeria: a case study of the vaccine direct delivery system

**DOI:** 10.1186/s12889-023-16575-x

**Published:** 2023-09-01

**Authors:** Ryoko Sato, Loveth Metiboba, Jamil Aliyu Galadanchi, Mohammed-Faosy Adeniran, Sadiq Haruna Hassan, David Akpan, Juliet Odogwu, Busayo Fashoto

**Affiliations:** 1 Harvard T.H. Chan School of Public Health, MA Boston, USA; 2 eHealth Africa, Abuja, Nigeria

**Keywords:** Cost analysis, Vaccine stockouts, eHealth technology, Vaccine direct delivery, Nigeria

## Abstract

**Introduction:**

Vaccine stockout is a severe problem in Africa, including Nigeria, which could have an adverse effect on vaccination coverage and even health outcomes among the population. The Vaccine Direct Delivery (VDD) program was introduced to manage vaccine stockouts using eHealth technology. This study conducts a cost analysis of the VDD program and calculates the incremental costs of reaching an additional child for vaccination through the VDD program.

**Methods:**

We used the expense reports from eHealth Africa, an NGO which implemented the VDD program, to calculate the VDD program’s overall operating costs. We also used the findings from the literature to translate the effect of VDD on the reduction of vaccine stockouts into its effect on the increase in vaccination coverage. We calculated the incremental costs of reaching an additional child for vaccination through the VDD program.

**Results:**

We calculated that implementing the VDD program cost USD10,555 monthly for the 42 months that the VDD program was operating in Bauchi state. This figure translates to an incremental cost of USD20.6 to reach one additional child for vaccination.

**Discussion/Conclusions:**

Our study is one of the first to conduct a cost analysis of eHealth technology in Africa. The incremental cost of USD20.6 was within the range of other interventions that intended to increase vaccine uptake in low- and middle-income countries. The VDD program is a promising technology to substantially reduce vaccine stockout, leading to a reduction of over 55% at a reasonable cost, representing 26% of the total budget for routine immunization activities in Bauchi state. However, there is no comparable costing study that evaluates the cost of a supply chain strengthening intervention. Future studies should investigate further the feasibility of eHealth technology, as well as how to minimize its costs of implementation while keeping the efficacy of the program.

**Supplementary Information:**

The online version contains supplementary material available at 10.1186/s12889-023-16575-x.

## Introduction

Vaccine stockouts are a universal problem [1], but the incidence of vaccine stockouts is particularly severe in sub-Saharan Africa. Lydon et al. (2017) found that 38% of sub-Saharan countries experience national-level stockouts. Reasons for stockouts are found to be predominantly internal, such as funding delays and poor stock management [2].

Studies have found that vaccine stockouts are one of the main barriers to vaccination worldwide [3, 4], including in Nigeria [5], the focus of the study. Recommendations of the Nigeria National Routine Immunization Strategic Plan in 2015 included the direct delivery of vaccines through transport companies to health facilities with appropriate cold chain storage capacity to mitigate the potential adverse effects of vaccine stockouts [6].

Despite the potentially severe and negative impact of vaccine stockouts on vaccination coverage as well as on long-term health outcomes, we have scarce evidence on how to mitigate vaccine stockouts. Iwu et al. (2019) found that only three studies were conducted to evaluate interventions for vaccine stock management in low- and middle-income countries, and those studies had limitations in the analytical methods [7].

In the context of Nigeria, Aina et al. (2017) found a positive effect of a logistic intervention on vaccine stockouts in Kano state, one of the north-central states in Nigeria [8]. Sato et al. (2021) conducted a causal study to find that the Vaccine Direct Delivery (VDD) program, which provided logistic support to each health facility, and which can support health facilities with stock management through eHealth technology, mitigated the monthly prevalence of vaccine stockouts as well as increased the number of vaccine doses administered in Bauchi state, a northern Nigerian state [9].

Digital health innovations (also described as “eHealth technology”) have become increasingly important in strengthening health systems in sub-Saharan Africa [10]. While eHealth technology is rapidly expanding in Africa, it faces various constraints, such as the shortage of trained staff and poor infrastructure [10]. It is important to strengthen the quality of human resources and infrastructure for the population in Africa to fully benefit from eHealth technology.

One obvious implication of eHealth technology due to the limited capacity is its cost aspect. We can easily imagine that the cost of implementing eHealth technology would be high in places where the infrastructure for it is not sufficiently established. Cost analyses would be useful to inform stakeholders how much it would cost to implement eHealth technology in African contexts. Nonetheless, cost studies around eHealth technology in Africa or in developing countries have been extremely limited [11]. Thus, this paper conducts a cost analysis of an innovative eHealth technology, the VDD program, and calculates the incremental cost of reaching one additional child for vaccination through the VDD program.

## Methods

### The Vaccine Direct Delivery (VDD) project

The Vaccine Direct Delivery (VDD) project was developed by eHealth Africa (eHA), a nongovernmental organization (NGO). Its objective was for the required stock of vaccines in health facilities to be delivered from state cold stores in a timely manner through eHealth technology to ensure sufficient stock at the facility at any given time.

The VDD project was based on an application called LoMIS Deliver that eHA developed, which enables stakeholders at all levels to plan, schedule, monitor, and manage the movement of health commodities, including vaccines, in near-real time. The VDD targets nine essential vaccines: BCG, measles, yellow fever, OPV, IPV, PCV, tetanus, HBV, and pentavalent [9].

Operationally, under VDD, eHA was responsible for maintaining the cold chain, distributing vaccines, diluents, and immunization cards from the zonal cold store to cold stores in local government areas (LGAs) and to health facilities which have refrigeration facilities for vaccine storage at the ward level [8].

In July 2015, eHA launched the VDD project in Bauchi state in collaboration with the State Primary Health Care Development Agencies (SPHCDA), the Bill and Melinda Gates Foundation (BMGF), the Dangote Foundation, other donors, and implementing partners. The VDD project in Bauchi state was operational for 42 months from July 2015 to December 2018, after which the project was transitioned to the Bauchi state government. The VDD project involved health facilities with functional cold chain equipment. See Sato et al. (2021) for the details on the VDD project [9].

Bauchi state is the fifth-most populous state among 36 states in Nigeria as of 2019, with over 7.5 million people [12]. Bauchi state has 20 LGAs in total and 284 primary health care facilities with functional cold chain equipment. Bauchi state struggles with a low vaccination rate: 28% of children aged 12 to 23 months did not receive any vaccination [13]. Only 36% of them received measles vaccine as of 2018 [13], while the measles burden remains high in Bauchi state [14]. The prevalence of vaccine stockouts in the state is high [15], and it impacts vaccination coverage negatively [16].

### Cost evaluation methodology: total costs

To calculate the overall costs of operating the VDD program, we used the expense reports from eHealth Africa. There were broadly four categories of costs: personnel costs, vaccine transportation costs, technical (software) support costs, and other program-related costs (Table 1).

**Table 1:** Categories of implementation costs of VDD

Category	Sub-category
Personnel	Drivers
	Delivery Coordinator
	Program Manager
	Project Manager
	Finance support staff
	GIS (geographic information system) support
	Software support
	Fringe
Vaccine transportation	Fueling
	Servicing and repairs
	Vehicle license
	Training for drivers
	Vehicle insurance
	Insurance
Software support	License and support
Other	Office Rent
	Phone allowance for personnel
	Internet usage
	Laptop maintenance
	Tablet maintenance
	Office supplies
	Miscellaneous

Personnel costs were required to monitor and ensure the smooth operation of the VDD program. Because one of the main features of the VDD program is to deliver vaccines, the transportation costs of vaccine delivery (vehicle-related costs) were an essential component of the VDD program. VDD software support costs were the technical maintenance costs for the VDD program. Lastly, other program-related costs included various components that were required to operate the VDD program, such as the office space and communications (phone and internet).

Our analysis mainly focuses on the implementation costs of VDD. Our cost categories in Table 1 do not include the development costs of VDD or capital costs because the eHealth technology and equipment used for the VDD program have also been used for many other programs that eHealth operates, and thus, such development and capital costs should be shared with all other programs that utilize this technology. However, we also present the development and capital costs of the VDD program in the Results section as a reference (See Appendix 1).

To derive the total costs of VDD’s implementation, we first calculated the monthly implementation cost of the VDD program, based on the actual expense incurred in 2019. Prior to 2019, expense reports were not made consistently – for example, some personnel costs were included in the other costs between 2015 and 2017, but they were later correctly categorized into personnel costs. The expense report in 2019 was the most comprehensive and complete; thus, we used the information from 2019 and assumed that the monthly cost derived based on the cost in 2019 is applicable to the years that VDD was operational between 2015 and 2018, after we controlled for inflation. Some costs were shared with other projects that eHA managed, such as office rent and internet use. For these items, we have taken the proportion of the cost, based on the number of projects eHA had. On average, eHA had 14 active projects at a given time. The expenses were reported in Naira, the Nigerian currency. We have used the conversion rate of 1 USD = 360 Naira [17].

In particular, the monthly implementation cost was first multiplied by 12 months to get the annual cost for 2016 to 2018. For 2015, because the project started in July, the monthly cost was multiplied by 6 months. Then, for each year, the inflation rate was applied. We used the annual inflation rate based on the consumer price index in Nigeria, available on the World Bank data page [18]. The inflation rate was 9% (2015), 15.7% (2016), 16.5% (2017), 12.1% (2018), and 11.4% (2019). Finally, we summed up the annual cost from 2015 to 2018 to derive the total operational costs of the VDD project in Bauchi state from July 2015 to December 2018.

### Cost evaluation methodology: cost per additional vaccination

In addition to calculating the overall implementation costs of the VDD program, we also derived the incremental costs required for the VDD program to vaccinate one additional child.

Here, we describe the steps for the calculation. First, we used the result from Sato et al. (2021) for the effect of VDD on the reduction of vaccine stockouts (Step 1) [9]. Sato et al. (2021) used the interrupted time series analysis method to evaluate the effect of VDD when it was operational from 2015 to 2018 in Bauchi state [9]. Table 2 presents their estimate of VDD’s effect. They found that in an average month, VDD reduced vaccine stockouts by 21.7% points. We divided the total implementation costs of VDD by this effect size to derive the unit cost to reduce vaccine stockout by 1% point. We assume that costs have a linear relationship with the reduction in vaccine stockout.

**Table 2:** Effect of VDD program on vaccine stockouts in Bauchi state, Nigeria

	Before VDD	After VDD	Difference	Difference (*p* = value)
	(1)	(2)		(3)
Stockout (any vaccines, 0/1)	0.389	0.172	0.217	0.000

Source: Sato et al. (2021) [9].

Second, we calculated the effect of the VDD program on the increase in vaccination coverage (Step 2). To do this, we first translated the effect of vaccine stockouts to the reduction of vaccination coverage. Table 3 presents the calculation process.

**Table 3:** Parameters used to calculate the effect of VDD on the increase in vaccine uptake

Item	Size	Notes / Interpretation
Effect of weekly stockout on the reduction of # children vaccinated against measles, per month, per LGA	77	Based on the result from Gooding et al. (2019) [14]. The effect of monthly stockout = 308 (77 × 4 weeks)
Total Number of measles vaccines given in Nigeria, per month	508,052	Source: DHIS2 data
Total number of LGAs in Nigeria	774	Source: aboutnigerians.com
Number of vaccines per LGA, per month	656	= 508,052/774
Reduction of # vaccines given due to a one-month stockout in a LGA (%)	0.469	= 308/656

We referred to the result from Gooding et al. (2019) for this conversion [16]. Based on data from 2015 to 2016, they found that weekly stockout of measles vaccine resulted in a reduction of 77 children vaccinated against measles per LGA per month on average across Nigeria. We converted this effect size to the effect of monthly stockout by multiplying it by 4 weeks to derive that a month-long stockout would result in a reduction of 308 children (77 × 4 = 308) receiving the vaccine per LGA per month. The VDD program intended to reduce the stockouts of not only measles vaccine, but 9 vaccines as mentioned above. We assumed that the effect of the vaccine stockouts on the measles vaccination from Gooding et al. (2019) could be applied to other vaccines as well [16].

Across Nigeria, 508,052 doses of measles vaccines were given per month in 2019, based on the data from the DHIS2 (District Health Information System 2) data platform. Because Nigeria has 774 LGAs in total, we calculated that each LGA distributes measles vaccine to 656 children per month. Note that the average number of vaccines distributed per LGA per month was calculated across Nigeria, not only in Bauchi state. This is because Gooding et al. (2019) derived the effect of stockout on the reduction of vaccinated children using national-level data [16].

From these calculations, we would estimate that a month-long stockout would result in the reduction of the number of vaccinated children by 308 from 656, which is equivalent to a 46.9% reduction in one LGA.

Now that we have the estimate of the volume reduction of vaccines given due to the stockout, we can calculate the effect of the VDD program on the increase in the likelihood of vaccine uptake by using information from both Tables 2 and 3, in particular by comparing the prevalence of stockout with and without VDD.

In Step 3, we converted the effect of the VDD program on the increased likelihood of vaccine uptake to the actual number of additional children who received vaccines due to the VDD program in Bauchi state. The total projected population in Bauchi state is 8 million (Table 4). We assume that the vaccine-eligible children are 24 months or below [19]. The percentage of the total population under 4 years old is 8% [20], and we assume that the proportion of the population under 2 years old (24 months) is half of the population under 4 years old, i.e., 4%. As a result, we calculated that the number of vaccine-eligible children in Bauchi is 320,000. Among these eligible children, 80.8% received some vaccinations, based on the Nigeria Demographic and Health Survey conducted in 2018 [13]. Thus, the total number of vaccinated children is 258,560. Using the effect size of the VDD program on the increase in the vaccine uptake calculated in Step 2, we derived the total number of additional children who were vaccinated due to the VDD program.

**Table 4:** Parameters used to calculate the actual number of children receiving a vaccine due to VDD

Item	Size	Notes
Total population in Bauchi state	8,000,000	Projected population based on the census data
% total population under 2 years old	4%	The percentage of total population under 4 years old is 8% [20] Assume the proportion of the population under 2 is half of the population under 4 years old.
Number of the vaccine-eligible children in Bauchi	320,000	= 8mil x 4%
Number of children receiving vaccine in Bauchi	258,560	80.8% of children received at least one vaccine [13] = 320,000 × 80.8%

Finally, we calculated the incremental costs required for the VDD program to vaccinate one additional child by dividing the total implementation costs of the VDD program by the number of additional children receiving a vaccine due to the VDD program.

## Results

Table 5 presents the monthly implementation costs of the VDD program by category. In total, it cost USD10,555 to implement the VDD program monthly. Almost half the total costs (46.3%) were personnel costs (USD4,891), followed by software support costs (21.3%, USD2,244), other program-related costs (17.0%, USD1,792), and vaccine transportation costs (15.4%, USD1,628). After adjusting for inflation each year, we derived that the total implementation cost of the VDD project from July 2015 to December 2018 was USD542,132.

**Table 5:** Monthly implementation costs of VDD program by category

Category	Sub-category	Costs (USD) in 2019
Personnel	Drivers	4891
	Delivery Coordinator	
	Program Manager	
	Project Manager	
	Finance support staff	
	GIS support	
	Software support	
	Fringe	
Vaccine transportation	Fueling	1628
	Servicing and repairs	
	Vehicle license	
	Training for drivers	
	Vehicle insurance	
	Insurance	
Software support	License and support	2244
Other	Office Rent	1792
	Phone allowance for personnel	
	Internet usage	
	Laptop maintenance	
	Tablet maintenance	
	Office supplies	
	Miscellaneous	

Because VDD reduced vaccine stockouts by 21.7% points (Table 2) and the VDD program cost USD542,132 in total in Bauchi state (Table 4), we derived that it cost USD24,983 to reduce vaccine stockout by 1% point.

Table 6 presents the effect of the VDD program on the increase in vaccine uptake. Based on Tables 2 and 3, we calculated the effect of the VDD program on the increase in vaccine uptake by comparing the prevalence of stockout with and without VDD. If the VDD program did not exist, stockouts would have occurred 38.9% of the time each month (Table 2), reducing vaccine uptake by 18.2% points overall, while the frequency of stockouts reduced to 17.2% of the time each month after the introduction of the VDD program (Table 2), which resulted in an 8.1-percentage-point reduction in vaccine uptake due to stockouts. Combining these results, we estimated that the VDD program increased vaccination coverage by 10.2% points.

**Table 6:** Effect of VDD on the increase in vaccine uptake

Item	Size	Notes / Interpretation
Effect of a monthly vaccine stockout on the reduction in vaccine uptake, without VDD (percentage points)	0.182	Assuming a month-long stockout happens 38.9% of time (base stockout rate from our result Table 2) The base-level stockout will cause 18.24% points decrease in the vaccine uptake (= 0.469 × 0.389)
Effect of a monthly vaccine stockout on the reduction in vaccine uptake, with VDD (percentage points)	0.081	Assuming a month-long stockout happens 17.2% of time (ex-post stockout rate from our result Table 2) The post-VDD stockout will cause 8.07% points decrease in the vaccine uptake (= 0.172 × 0.389)
The increase in the vaccine uptake due to VDD (percentage points)	0.102	Base stockout rate – Ex-post stockout rate (= 0.182 − 0.081)

Table 7 presents the calculated number of additional children who received the vaccine due to VDD as well as the incremental costs required for the VDD program to vaccinate one more child in Bauchi state. Using the data on the total eligible population in Bauchi state (Table 4) and the result from Table 6, we calculated that the number of additional children receiving vaccines due to the VDD program was 26,373. Because the VDD program cost USD542,132 in total in Bauchi state, we derived that it cost USD20.6 for the VDD program to vaccinate one additional child.

**Table 7:** Incremental costs required to vaccinate one additional child

Item	Size	Notes
Additional # children receiving vaccines due to VDD program	26,373	If VDD increases the vaccine uptake by 10.2% points (From Table 6). Total # vaccinated child was 258,560. Additional # vaccinated children = 258,560 × 10.2% points.
Costs required to reach one additional child for vaccine due to VDD program ($)	20.56	= USD542,132/26,373

As a reference, we also calculated the development and capital costs. Based on the proportion of the monthly salaries of software developers dedicated to developing the application LoMIS Deliver between 2016 and 2018, it is estimated that the development costs were $101,552 (Appendix 1). Fixed capital costs totaled $134,622, after taking into account depreciation and converting the costs for the 42 months that the VDD program was operational in Bauchi state.

## Discussion

This paper conducted a cost analysis of the VDD program, an innovative eHealth technology to reduce vaccine stockouts, in Bauchi state, Nigeria.

The purpose of this study is to inform policymakers and stakeholders of the costing aspect of an eHealth technology that has been proven successful in reducing vaccine stockouts in the African context, as the scientific evidence on the cost analysis of eHealth technology is extremely scarce, especially in developing countries. Cost analysis of eHealth technology is especially important in the African context because recent years have witnessed substantial eHealth innovation in the region, and it is increasingly becoming a norm to rely on it for health system management [21].

We found that implementation of the VDD program cost USD10,555 monthly and USD542,132 for the 42 months when VDD was operating in Bauchi state. This translates to a cost of USD24,983 to reduce vaccine stockout by 1% point from the baseline stockout rate of about 40%.

The major components of the implementation costs were personnel costs (46%) and software support costs (21%). On average, it cost USD20.6 for the VDD program to vaccinate one additional child.

According to a paper that conducted a systematic literature review on the effectiveness of interventions to increase vaccination coverage in low- and middle-income countries in 2019, interventions cost between $0.46 and $111.8 per vaccinated child in 2019 USD [22]. In this systematic review, there were four studies that were conducted on or after 2010, and the two most recent studies had higher costs per vaccinated child: $28.6 and $111.8, respectively. While our cost estimate of the VDD program might be comparable to some of these existing studies, we did not find any paper that evaluated the incremental costs of supply chain strengthening interventions, which could be appropriate programs to which we could compare VDD.

In 2017, Bauchi state allocated 250 million naira for routine immunization activities (values in 2017 Naira) [23]. This is equivalent to about USD39,930 monthly budget in 2019 USD. Because the monthly cost of the VDD program was USD10,555, the VDD program would represent about 26% of the total costs of routine immunization activities, with the potential to reduce stockouts by over 20% points, or more than a 55% reduction from the baseline stockout rate.

In Nigeria, where child mortality due to vaccine-preventable diseases is high and vaccination coverage remains low, with a persistent problem of unstable vaccine supply, the VDD program offers an innovative and practical solution at a reasonable cost to provide sustainable vaccine supply and eventually to reduce child mortality by improving vaccination coverage.

As a future study, we could strategize how to further mitigate costs while keeping the VDD program as effective as possible. In particular, because our analysis identified that the personnel costs and software support costs were the main cost components of the VDD program, we might consider how to reduce such costs. For example, it is essential to coordinate with the national immunization programs, such as EPI (Expanded Program on Immunization) and SIA (Supplementary Immunization Activities), and possibly integrate the VDD into these programs, as these are the most critical times for assuring the vaccine stock.

This study had several limitations. First, the category of expense records kept at eHA was not consistent over time, and thus, we needed to use the expense incurred in a particular year (2019) and adopt it in other years. Second, there was no direct information on the actual number of children who benefited from vaccination due to the VDD program. This is partially because the accurate number of population in Nigeria is not available: the last national census was conducted in 2006, and any population estimate is based on this census that was implemented more than 15 years ago. Third, we assumed that costs have a linear relationship with the reduction in vaccine stockouts in order to derive the costs required to reduce vaccine stockouts. In reality, reducing vaccine stockouts at a small scale might cost almost as much as reducing stockouts at a larger scale due to the initial setup cost. Fourth, the effect of stockouts on the reduction of vaccination was drawn from Gooding et al. (2019), which was the average effect across all the LGAs in Nigeria, but not specific to Bauchi state. Given that the vaccination coverage in Bauchi state is lower than the national average – for example, measles coverage was 35.5% in Bauchi state, while the national average was 54% (NDHS, 2018) – the effect of stockouts in Bauchi state might be different from that at the national level. Fifth, this is one of the first costing studies of eHealth technology in Africa, and there is virtually no other study that conducted a similar costing analysis for eHealth technology. While this makes the paper unique and valuable, it is a limitation in the sense that there is no study to compare the cost-effectiveness to.

## Conclusion

Our study is one of the first to conduct a cost analysis of a successful eHealth technology in Africa, the VDD program, which reduces vaccine stockouts and improves vaccination coverage. The monthly implementation cost of the VDD program was USD10,555, and it cost USD24,983 to reduce vaccine stockout by 1% point. The incremental cost for the VDD program to vaccinate one additional child was USD20.6. The VDD program provided an innovative and practical solution to substantially reduce vaccine stockouts at a reasonable cost, representing 26% of the total budget for routine immunization activities within the state. A future study could investigate how to minimize the operational costs while keeping the efficacy of the program.

### Supplementary Information

**Additional file 1.**

## Data Availability

The datasets generated and/or analyzed during the current study are not publicly available because they are the property of eHA but are available from the corresponding author on reasonable request.

## Abbreviations

VDD: Vaccine Direct Delivery
BCG: Bacillus Calmette–Guérin vaccine
OPV: Oral Polio vaccine
IPV: Inactivated Poliovirus vaccine
PCV: Pneumococcal Conjugate vaccine
HBV: Hepatitis B vaccine
eHA: eHealth Africa
LGA: Local government area
NGO: Non-governmental organization
SPHCDA: State Primary Health Care Development Agencies
BMGF: Bill and Melinda Gates Foundation
GIS: Geographic information system
EPI: Expanded program on immunization
SIA: Supplementary immunization activities
