# Supplementary material for: Cost analysis of an innovative eHealth program in Nigeria: a case study of the vaccine direct delivery system
Source: BMC Public Health. 2023 Sep 1;23:1691. doi: 10.1186/s12889-023-16575-x (PMC10472608; doi:10.1186/s12889-023-16575-x)
Supplement: Supplementary file 1 — Additional file 1. [file 12889_2023_16575_MOESM1_ESM.docx]

Calculation of Development and Capital costs

Development Costs

Development costs included the proportion of monthly salary of software developers dedicated to developing the application, LoMIS Deliver, between 2016 and 2018. Below is the breakdown of the development costs.

| Description | Amount (USD) | note |
| --- | --- | --- |
| Proportion of monthly salary charged to the development of LOMIS Deliver (2016) | 49,685 | In 2016, LoMIS Deliver was used in 3 states: Kano, Bauchi and Sokoto. Thus the development cost is divided by 3 |
| Proportion of monthly salary charged to the development of LOMIS Deliver (2017) | 19,357 | In 2017, LoMIS Deliver was used in 3 states: Kano, Bauchi and Sokoto. Thus the development cost is divided by 3 |
| Proportion of monthly salary charged to the development of LOMIS Deliver (2018) | 32,511 | In 2017, LoMIS Deliver was used in 2 states: Bauchi and Sokoto. Thus the development cost is divided by 2 |
| Total | 101,552 |  |

Fixed Capital Costs

There were fixed capital costs incurred for the VDD program. There were broadly 3 categories: vehicle, cold chain equipment, and office supplies. We assumed that the life expectancy of these items was 8 years, and 8% depreciation rate. Below is the breakdown of the capital costs:

| Description | total costs (USD) | Value for 42 months (USD) |
| --- | --- | --- |
| Vehicles | 210076 | 124,726 |
| Cold chain equipment | 15659 | 9297 |
| Office supplies | 1010 | 600 |
| Total |  | 134,622 |
